# Correlation analysis of cognitive dysfunction and fluctuations in serum fibroblast growth factor 21 (FGF21) levels in the overweight group

**DOI:** 10.5937/jomb0-59295

**Published:** 2026-02-27

**Authors:** Guotian Lyu, Xiaoli Lu, Qichang Meng, Yanyan Liu, Shuyan Liu, Jieyi Liu, Peng Dong

**Affiliations:** 1 Dongyang Hospital of Traditional Chinese Medicine, Department of Neurology, Dongyang City, China; 2 Dongyang Hospital of Traditional Chinese Medicine, Endoscopy Centre's Office, Dongyang City, China; 3 Peking University First Hospital, Department of Endocrinology, Beijing City, China; 4 The People's Hospital of Danyang (Affiliated Danyang Hospital of Nantong University), Department of Neurology, Danyang City, China; 5 No. 905 Hospital of People's Liberation Army Navy Affiliated to Naval Medical University, Department of Neurology, Shanghai City, China

**Keywords:** obese teenagers, cognitive function, behavioural test, fibroblast growth factor 21 (FGF21), gojazni adolescenti, kognitivna funkcija, bihevijoralni testovi, fibroblastni faktor rasta 21 (FGF21)

## Abstract

**Background:**

To explore the changing characteristics of cognitive function in overweight/obese (OWO) adolescents and analyse its relationship with the level of serum fibrob-last growth factor 21 (FGF21).

**Methods:**

A total of 175 adolescents were selected and divided into a normal body mass index (BMI) group (n = 50), an overweight BMI group (n = 50), and an obese BMI group (n=75). All participants underwent assessment of anthro-pometric indicators (height, weight, waist circumference, BMI Z score). Fasting venous blood was collected to meas-ure the level of serum FGF21 (by enzyme-linked immuno-sorbent assay (ELISA)), as were metabolic parameters such as fasting plasma glucose (FPG), fasting insulin (FINS), gly-cated haemoglobin (HbA1c), and lipid profiles (TC, TG, LDL-C, HDL-C). Overall cognitive function was evaluated via the Chinese version of the Montreal Cognitive Assess-ment Foundation Scale (MoCA-B), executive function was assessed via the Wisconsin Card Sorting Test (WCST) (with a focus on analysing the number of persistent errors/PE and the number of completed classifications/CC), and working memory was evaluated via the number span test (DST). Independent sample t tests or Mann Whitney U tests were used to compare the differences between groups. Pearson or Spearman correlation analysis was used to explore the relationships between serum FGF21 levels and cognitive indicators and metabolic parameters. Multiple linear regres-sion was used to analyse the independent association between serum FGF21 and cognitive function scores (after adjusting for potential confounding factors such as age, sex, BMI Z score, and HOMA-IR).

**Results:**

Compared with those of normal individuals, the sys-to-lic blood pressure, diastolic blood pressure, fasting blood glucose, glycated haemoglobin and triglyceride levels of ado-lescents in the obese group were greater (all P&lt;0.05). Under the consistent or inconsistent stimulation conditions of the Flanker task, there was no statistically significant differ-ence in the ACC between any two groups of adolescents. Compared with those in the normal body type group and the overweight group, the reaction time of adolescents in the obese group was prolonged (all P&lt;0.05). In the n-back task, there was no statistically significant difference in the ACC between any two groups of adolescents. However, the response time of adolescents in the obese group in the 1-back and 2-back tasks was longer than that in the normal body type group and the overweight group (all P&lt;0.05). Compared with those in the normal body type group, the serum FGF21 levels of adolescents in the obese group were greater (P=0.001). The results of the partial correlation analysis revealed that the reaction time of adolescents in the Flanker and n-back tasks was correlated with their BMI, body fat mass, waist circumference, waist hip ratio, FGF21 level, etc. (all P&lt;0.05). Multiple linear regression analysis further confirmed that BMI was associated with prolonged response time in cognitively related behavioural tasks in adolescents (all P&lt;0.05), and the level of FGF21 was correlated with the ACC in the 2-back task (P=0.001) and the response time to inconsistent stimuli (P=0.048).

**Conclusions:**

Overweight adolescents have significant cogni-tive impairment, with significantly elevated serum FGF21 lev-els, and elevated FGF21 levels are independently associated with poorer overall cognitive and executive functions.

## Abstract (sr)

**Uvod:**

Cilj je bio da se istraže promene u karakteristikama kognitivne funkcije kod gojaznih i prekomerno uhranjenih adolescenata i analizirati njihovu povezanost sa nivoom fibroblastnog faktora rasta 21 (FGF21) u serumu.

**Metode:**

Ukupno je odabrano 175 adolescenata koji su podeljeni u tri grupe: grupa sa normalnim indeksom telesne mase (BMI) (n = 50), grupa sa prekomernom telesnom težinom (n = 50) i grupa gojaznih (n=75). Svim učesnicima su procenjeni antropometrijski parametri (visi-na, težina, obim struka, Z skor za BMI). Uzorci venske krvi u stanju gladovanja su uzeti za merenje nivoa FGF21 u serumu (ELISA metodom), kao i metaboličkih parametara kao sto su glukoza u plazmi na prazan stomak (FPG), insu-linemija u stanju gladovanja (FINS), glikozilirani hemoglo-bin (HbA1c) i lipidni profil (TC, TG, LDL-C, HDL-C). Opsta kognitivna funkcija je procenjena je pomocu kineske verzije Montrealske skale za kognitivnu procenu (MoCA-B), izvrsna funkcija pomocu Viskonsin testa za sortiranje kartica (WCST) (posebno analiziran broj uporno ponovljenih gresaka/PE i broj zavrsenih klasifikacija/CC), dok je radna memorija procenjena testom niza brojeva (DST). Za poređenje razlika između grupa korisćeni su t-testovi za nezavisne uzorke ili Mann-Whitney U testovi. Pearsonova ili Spearmanova korelaciona analiza korišćena je za ispitivanje odnosa između nivoa FGF21 u serumu i kognitivnih pokazatelja i metaboličkih parametara. Visestruka linearna regresija korišćena je za analizu nezavisne povezanosti između nivoa FGF21 u serumu i rezultata kognitivnih testo-va (uz kontrolu potencijalnih faktora kao sto su uzrast, pol, Z skor za BMI i HOMA-IR).

**Rezultati:**

U poređenju sa adolescentima normalne telesne mase, adolescenti u gojaznoj grupi imali su vise vrednosti sistolnog i dijastolnog krvnog pritiska, glukoze u krvi na prazan stomak, glikoziliranog hemoglobina i triglicerida (sve P&lt;0,05). U uslovima konzistentne i nekonzistentne stimulacije u Flanker zadatku nije bilo statistički značajnih razlika u tačnosti (ACC) između bilo koje dve grupe ado-lescenata. Međutim, vreme reakcije adolescenata u gojaznoj grupi je bilo produženo u poređenju sa normal-nom i prekomerno uhranjenom grupom (sve P&lt;0,05). U n-back zadatku takođe nije bilo statistički značajne razlike u ACC između grupa, ali je vreme reakcije u 1-back i 2-back zadacima bilo duže kod adolescenata u gojaznoj grupi (sve P&lt;0,05). Nivoi FGF21 u serumu bili su značajno visi kod adolescenata u gojaznoj grupi u poređenju sa onima u grupi normalne telesne mase (P=0,001). Rezultati parci-jalne korelacione analize pokazali su da je vreme reakcije u Flanker i n-back zadacima bilo u korelaciji sa BMI, masom telesne masti, obimom struka, odnosom struk-kuk i nivoom FGF21 (sve P&lt;0,05). Visestruka linearna regresiona analiza dodatno je potvrdila da je BMI povezan sa produženim vremenom reakcije u kognitivnim zadacima kod adolescenata (sve P&lt;0,05), a nivo FGF21 bio je povezan sa ACC u 2-back zadatku (P=0,001) i vremenom reakcije na nekonzistentne stimuluse (P=0,048).

**Zaključak:**

Adolescenti sa prekomernom telesnom težinom i gojaznosću pokazuju značajne kognitivne poremećaje, uz povisene nivoe FGF21 u serumu. Poviseni nivoi FGF21 su nezavisno povezani sa losijim rezultatima u zadacima opste i izvrsne kognitivne funkcije.

## Introduction

Overweight and obesity are increasingly serious global health problems. Epidemiological surveys have shown that the prevalence of overweight and obesity among children and adolescents aged 6-17 years has continued to rise from 2020-2024, with an overweight rate of 11.1% and an obesity rate of 7.9% [1]. Research [2] has shown that obese patients are at greater risk of developing type 2 diabetes, cardiovascular and cerebrovascular diseases, hypertension and other diseases, which places greater medical and economic burdens on families and society.

Cognitive dysfunction manifests as a reduction in cognitive ability in one or more cognitive domains, such as language, memory, and reasoning [3]. Previous studies have shown through the Central Nervous System Vital Signs (CNSVS) scale that the scores of obese adolescents in aspects such as memory and executive function are significantly lower than those of healthy adolescents [4], suggesting a decline in cognitive function in obese adolescents. Furthermore, studies [5] have reported that, compared with children in the control group, obese children also have deficiencies in attentional transition function. Compared with cognitive-related scales, behavioural tests are more sensitive in the assessment of cognitive function [6]. The Flanker task, also known as the lateral inhibition task, requires subjects to respond to the target stimulus while suppressing the influence of irrelevant stimuli, which can reflect an individual's ability to process cognitive conflicts [7]. Consistent stimuli are used to evaluate basic cognitive functions, and inconsistent stimuli are used to evaluate higher-order cognitive functions [8]. The Flanker task has relatively wide application in research on executive function and is a suitable tool for assessing cognitive control. However, its application in research on cognitive control in adolescents is relatively limited [9]. The n-back task, also known as the reciprocal N-item test paradigm, can reflect an individual's ability to store and process information over a short time. Among them, the 0-back task is used to evaluate immediate memory, and the 1-back and 2-back tasks are used to evaluate delayed memory [10]. This task can also be used to evaluate an individual's working memory, which plays an important role in reading comprehension, reasoning, arithmetic calculation, solving mathematical problems, and improving academic performance [11]. However, at present, systematic cognitive assessment studies using behavioural tests for overweight and obese adolescents are still relatively rare.

Studies have shown that the connection between cognitive-related diseases and obesity may be mediated by the pleiotropic effects of endocrine factors, especially some endocrine factors secreted by metabolism-related tissues and organs (such as leptin, adiponectin and fibroblast growth factor 21 (FGF21)). It can act on the central nervous system through the blood-brain barrier and participate in the occurrence and development of cognitive-related diseases [12]. FGF21 is a hormone-like endocrine factor that regulates glycolipid metabolism and energy homeostasis in the body and is expressed mainly in tissues such as the liver and fat. Moreover, FGF21 can be secreted and produced by multiple tissues throughout the body and exerts neuroprotective effects. It can be produced in small amounts by cells within the central nervous system (such as glial cells and neurons) to exert protective effects directly or can be produced by peripheral tissues and then cross the blood-brain barrier to enter the central nervous system to exert protective effects indirectly [13]. A previous study [14] revealed that FGF21 could improve the cognitive function of ageing mice induced by D-galactose by reducing hippocampal damage. However, in the population, especially among obese adolescents, the relationship between serum FGF21 levels and cognitive function remains unclear.

This study comprehensively evaluated the cognitive function of adolescents through behavioral tests (including the flanker task and the n-back task), analysed the correlations between metabolic indicators related to obesity in adolescents and cognitive function, as well as the influencing factors of overweight and obesity in adolescents on cognitive function, and explored the associations between serum FGF21 and the cognitive function of adolescents. To provide a new perspective for research on the mechanism of cognitive function changes caused by obesity.

## Materials and methods

### Research subjects and their groups

The inclusion criteria were as follows: age ranging from 15 to 18 years and stable body weight in the past 90 days (self-reported body weight change <5 kg). The exclusion criteria were as follows: having a history of weight loss drugs or having received surgical treatment for obesity; suffering from nonsimple obesity, including endocrine and metabolic diseases (such as Cushing's syndrome, hypothyroidism, hypogonadism, and polycystic ovary syndrome), tumors and trauma; having a history of severe mental illness (such as schizophrenia, bipolar disorder, and major depression); and suffering from bulimia nervosa disorder.

Ultimately, a total of 175 adolescents were included in this study. In accordance with the Health Industry Standard of the People's Republic of China - Screening for overweight and obesity in school-aged children and adolescents [15], the participants were divided into the obesity group (n = 75), the overweight group (n = 50), and the normal body type group (n = 50). Among them, the sex ratios and ages of the three groups of teenagers were matched.

### Recording and collection of data

The general information of the subjects was recorded as follows: (1) General demographic data, including age and sex. (2) Anthropometric data, including height, body weight, waist circumference, hip circumference, systolic blood pressure (SBP), diastolic blood pressure (DBP), and body composition data (body fat mass, body fat percentage), were obtained, and the body mass index (BMI) and waist hip ratio were calculated. Moreover, laboratory indicators, including fasting plasma glucose (FPB), fasting insulin (FINS), and glycosylated haemoglobin, were collected from the subjects. HbA1c, serum creatinine (Scr), uric acid (UA), alanine transaminase (ALT), aspartate transaminase (AST), glutamyl transpeptidase (GGT), total cholesterol (TC), triacylglycerol TAG, high-density lipoprotein cholesterol (HDL-Ch), and low-density lipoprotein cholesterol (LDL-Ch) levels.

### Behavioural test methods

Behavioural tests were used to evaluate the cognitive function of the adolescents. This experiment needs to be completed in the school's computer classroom. The environment should be kept quiet, and all the subjects should sit at intervals with partitions in between. The subjects were school teachers who had received standardised training in behavioural tests and were responsible for guiding the subjects to use E-Prime 3.0 software to participate in the behavioural tests. Before the experiment, the experimenter played a video explaining the rules for the subjects and answered their questions about the rules. After confirming that the subjects understood the rules through demonstration questions, the formal experiment began. The performance of behavioural tests includes two indicators: accuracy (ACC) and reaction time.

### Flanker mission

During the test, the subjects first saw »Countdown 3, 2, 1« on the computer screen. Then they saw the instructions »Please determine the direction of the middle arrow. Press 1 to the left and 2 to the right«, followed by seeing the consistent stimulus and inconsistent stimulus pictures. Each type of stimulus picture was presented for 120 seconds, after which the participants completed the task. The experimental process consisted of 2 blocks, each with 40 stimuli. The ratio of consistent stimuli to inconsistent stimuli was 1:1, the stimuli were presented in a random order, and the observation distance of the subjects was 1 m. Before the formal trial, the subjects needed to be trained to familiarise themselves with the trial scenario and reduce the practice effect.

### n-back task

During the test, the subjects first saw »Countdown, 3, 2, 1@ on the computer screen and then saw the instructions »Please determine whether the current letter is the same as the letter X (0-back)/the previous one (1-back)/the letter before last (2-back). If they are the same, press 1; if not, press 2«, and then see the letters. The presentation time of each group of blocks is 42 s, and each experiment is repeated three times. Finally, the conclusion is presented. The experimental process consists of a total of 9 blocks, and each block contains 12 stimuli. The observation distance of the subjects was 1 meter. Before the formal trial, the subjects needed to be trained to familiarise themselves with the trial scenario and reduce the practice effect.

### Detection of serum FGF21 levels

The serum FGF21 level of the subjects was detected via an enzyme-linked immunoassay kit (Antibody and Immunoassay Services, Hong Kong, China) [16], and the specific procedure was carried out in accordance with the kit instructions. The minimum concentration of FGF21 detectable by this kit is 30 pg/mL, and the differences within and between batches are 5.41% and 5.79%, respectively.

### Statistical methods

Statistical analysis was conducted via SPSS version 25.0 software. The quantitative data with a normal distribution are expressed as x±s, and the quantitative data with a nonnormal distribution are expressed as M (Q1, Q3). One-way analysis of variance was used for comparisons between groups. Among them, a logarithmic transformation was required for non-normally distributed data before analysis. Qualitative data are expressed as frequencies. The χ^2^ test was used for comparisons between groups. During the assessment of cognitive function, sex and age were corrected first to exclude their influence on cognitive function. Partial correlation analysis was used to evaluate the associations between cognitive function, anthropometric data, and laboratory test indicators. A multiple linear regression model was adopted to explore further the correlation between cognitive function and anthropometric data, as well as laboratory test indicators. P<0.05 indicated that the difference was statistically significant.

## Results

### Comparison of data from the three groups of subjects

Compared with those of the normal body type group, the body fat mass, body fat percentage, waist circumference, hip circumference, waist-hip ratio, SBP DBF, FINS, UA, ALT, and GGT levels of the overweight and obese groups tended to increase (all P=0.001). The levels of SBP, DBP FPG, HbA1c and TAG in the obese group were greater than those in the normal body type group (all P<0.05), and the level of HDL-Ch was lower than that in the normal body type group (P=0.045). The specific data are shown in Table 1.

**Table 1:** Comparison of general information and laboratory testing indicators among the three groups of subjects. Note: 1mmHg=0.133kPa.<br>* indicates the comparison with the normal weight group, P<0.05; # indicates the comparison with the overweight group, P<0.05.

Item	Normal weight group<br>(n=50)	Overweight group<br>(n=50)	Obese group<br>(n=75 )	P value
General information				
Age/year	15.18±0.48	15.52±0.58	15.31±0.52	0.327
Gender (male/female)/n	29/21	30/20	50/25	0.117
BMI/(kg-m^-2^)	21.35±2.16	25.26±0.98*	30.37±2.73*#	0.001
Body fat mass/kg	12.73±4.90	20.91±4.69*	29.95±6.62*#	0.001
Body fat percentage/%	20.93±7.73	28.47±7.21*	33.55±5.83*#	0.001
Waist circumference/cm	73.95±7.48	82.87±3.69*	95.53±9.14*#	0.001
Hip circumference/cm	94.70±5.77	103.71±3.80*	110.09±5.38*#	0.001
Waist-to-hip ratio	0.80±0.04	0.84±0.01*	0.89±0.03*#	0.001
SBP/mmHg	115.04±12.27	120.29±11.51	124.39±9.98*	0.001
DBP/mmHg	63.84±7.42	69.09±8.73*	70.44±9.27*	0.001
Laboratory testing indicator				
FPG/(mmol-L^-1^)	4.62±0.35	4.78±0.30	4.79±0.37*	0.014
FINS/(mmol-L^-1^)	15.03 (11.43, 19.28)	21.67 (17.62, 29.76)*	29.94 (18.96, 38.32 )*#	0.001
HbA1c/%	5.31±0.21	5.32±0.16	5.42±0.27*	0.008
Scr/(*μ*mol-L^-1^)	65.95 (58.40, 76.85)	66.25 (56.33, 73.83)*	71.90 (63.50, 79.50)*#	0.052
UA/(*μ*mol-L^-1^)	368.86±85.19	397.66±82.31	451.24±102.53*	0.001
ALT/(U-L^-1^)	14.00 (11.00, 17.25)	17.50 (15.00, 27.00)*	26.00 (20.00, 48.00)*#	0.001
AST/(U-L^-1^)	21.00 (17.75, 24.00)	21.00 (18.00, 26.25)	26.00 (20.00, 32.00)*	0.001
GGT/(U-L^-1^)	16.50 (14.00, 21.00)	19.50 (14.75, 25.00)	23.00 (17.00, 32.00 )*#	0.001
TC/(mmol-L^-1^)	4.36±0.78	4.30±0.76 *	4.33±0.74*#	0.88
TAG/(mmol-L^-1^)	0.74 (0.55, 1.04)	0.87 (0.61, 1.29)	0.92 (0.69, 1.37)	0.012
HDL-Ch/(mmol-L^-1^)	1.44±0.29	1.26±0.26*	1.21±0.25*	0.001
LDL-Ch/(mmol-L^-1^)	2.48±0.70	2.56±0.56	2.69±0.69	0.076

### Assessment of the cognitive function of the subjects

An analysis was conducted on whether the sex and age of the three groups of subjects affected their cognitive functions. The results revealed that there were no statistically significant differences in the ACC or response time between adolescents in the first year of high school (15.0-16.5 years old) and those in the second year of high school (16.5-18.0 years old) in any group. Moreover, among the 175 teenagers, there was no statistically significant difference in these two indicators between males and females. These findings suggest that age and sex do not influence performance in adolescent behavioural tests.

With respect to the ACC, there was no statistically significant difference among the three groups of subjects in the Flanker task or the n-back task. In terms of reaction time, the performance of the three groups of subjects differed: (1) Flanker task. Under conditions of consistent stimulation or inconsistent stimulation, the response time of the subjects in the obese group was longer than that in the normal body type group (P=0.000, P=0.002) and longer than that in the overweight group (P=0.030, P=0.001). However, there was no statistically significant difference between the normal body type group and the overweight group. (2) N-back task. With increasing memory load, the reaction time of the subjects in the three groups gradually increased. With the increase in the degree of obesity, the response time of the subjects in the 0-back task increased. In the 1-back task, the response time of the subjects in the obese group was longer than that of those in the normal body type group (P=0.000). In the 2-back task, the response time of the subjects in the obese group was not only longer than that of those in the normal body type group (P=0.000) but also longer than that of those in the overweight group (P=0.001). The specific data are shown in Table 2.

**Table 2:** Cognitive function assessment of the three groups of subjects.

Item	Normal weight group<br>(n=50 )	Overweight group<br>(n=50)	Obese group<br>(n=75 )	P value
Flanker task				
ACC				
Congruent stimulus	0.97±0.09	0.97±0.04	0.98±0.04	0.442
Incongruent stimulus	0.92±0.09	0.93±0.10	0.92±0.14	0.854
Reaction time/ms				
Congruent stimulus	406.12±108.32	431.35±85.17	475.13±84.31	0.001
Incongruent stimulus	465.41±95.59	463.55±59.92	524.01±103.79	0.001
n-back task				
ACC				
0-back	0.94±0.05	0.93±0.07	0.93±0.08	0.641
1-back	0.84±0.12	0.85±0.08	0.86±0.12	0.404
2-back	0.78±0.13	0.72±0.16	0.78±0.15	0.751
Reaction time/ms				
0-back	522.52±103.11	539.48104.85	564.34±116.74	0.05
1-back	541.76±149.67	587.90±160.04	656.60±168.99	0.001
2-back	570.49±174.48	625.08±199.08	754.24±201.57	0.001

### Relationships between the behavioural test performance of the subjects and their anthropometric data, as well as laboratory test indicators

After adjusting for sex and age, we used partial correlation analysis to evaluate the correlation between the cognitive function of the subjects and their anthropometric data and laboratory test indicators. (1) Regarding the ACC, there was no statistically significant difference among the three groups of subjects in the flanker task or the n-back task. (2) In terms of response time, the results of the Flanker task (Table 3) revealed that under consistent stimulation conditions, the response time of the subjects was correlated with their BMI, body fat mass, body fat percentage, waist hip ratio, waist circumference, and hip circumference (all P<0.05). Under inconsistent stimulation conditions, response time was only correlated with BMI, body fat mass, waist hip ratio, waist circumference, and Scr and UA levels (all P<0.05). The results of the n-back task (Table 4) revealed that, in the 1-back task, the response time of the subjects was correlated with their BMI; body fat mass; body fat percentage; waist hip ratio; waist circumference; hip circumference; and ALT, AST, GGT, and UA levels (all P<0.05). In the 2-back task, the response time of the subjects was correlated with their BMI; body fat mass; body fat percentage; waist hip ratio; waist circumference; hip circumference; and ALT, AST, and UA levels (all P<0.05).

**Table 3:** Correlation between the performance of subjects in the Flanker task and their anthropometric data and laboratory testing indicators r (P) value.

Item	ACC		Reaction time	
	Incongruent stimulus	Congruent stimulus	Incongruent stimulus	Congruent stimulus
BMI	-0.004 (0.958)	0.033 (0.678)	0.259 (0.001)	0.337 (0.001)
Body fat mass	0.004 (0.963)	0.014 (0.857)	0.154 (0.045)	0.242 (0.001)
Body fat percentage	-0.013 (0.874)	-0.024 (0.764)	0.061 (0.431)	0.193 (0.011)
Waist-to-hip ratio	0.008 (0.928)	0.054 (0.495)	0.217 (0.004)	0.280 (0.001)
Waist circumference	0.007 (0.930)	-0.018 (0.820)	0.156 (0.042)	0.269 (0.001)
Hip circumference	-0.016 (0.848)	-0.027 (0.731)	0.107 (0.162)	0.238 (0.002)
SBP	0.051 (0.538)	0.006 (0.940)	0.041 (0.596)	0.109 (0.155)
DBP	-0.029 (0.729)	0.034 (0.674)	-0.042 (0.581)	0.058 (0.452)
FINS	-0.053 (0.523)	0.030 (0.707)	0.088 (0.250)	0.132 (0.084)
HbA1c	-0.114 (0.168)	-0.037 (0.639)	0.023 (0.765)	0.110 (0.153)
FPG	0.072 (0.387)	0.012 (0.877)	-0.074 (0.336)	0.032 (0.680)
ALT	-0.105 (0.204)	0.041 (0.607)	0.100 (0.191)	0.142 (0.062)
AST	-0.099 (0.231)	0.069 (0.384)	0.115 (0.133)	0.099 (0.194)
GGT	-0.095 (0.251)	0.057 (0.473)	0.088 (0.251)	0.123 (0.107)
Scr	0.075 (0.363)	0.077 (0.335)	0.219 (0.004)	0.049 (0.521)
UA	-0.067 (0.420)	0.035 (0.662)	0.171 (0.025)	0.139 (0.069)
TC	0.026 (0.755)	-0.053 (0.508)	-0.015 (0.849)	-0.116 (0.130)
TAG	-0.112 (0.177)	-0.113 (0.154)	0.002 (0.975)	-0.003 (0.973)
HDL-Ch	-0.014 (0.863)	0.063 (0.425)	0.014 (0.855)	-0.045 (0.556)
LDL-Ch	0.042 (0.616)	-0.083 (0.293)	-0.005 (0.950)	-0.098 (0.202)

**Table 4:** Correlation between the performance of subjects in the n-back task and their anthropometric data and laboratory testing indicators r (P) value.

Item	ACC			Reaction time		
	0-back	1-back	2-back	0-back	1-back	2-back
BMI	0.032 (0.701)	0.078 (0.344)	0.087 (0.299)	0.151 (0.069)	0.268 (0.001)	0.351 (0.001)
Body fat mass	0.027 (0.745)	0.057 (0.489)	0.061 (0.465)	0.124 (0.135)	0.217 (0.004)	0.282 (0.001)
Body fat percentage	0.030 (0.722)	0.042 (0.615)	0.062 (0.459)	0.065 (0.438)	0.173 (0.023)	0.240 (0.001)
Waist-to-hip ratio	0.002 (0.983)	-0.022 (0.794)	0.077 (0.357)	0.095 (0.253)	0.257 (0.001)	0.320 (0.001)
Waist circumference	-0.058 (0.491)	0.001 (0.986)	0.008 (0.921)	0.122 (0.143)	0.200 (0.008)	0.248 (0.001)
Hip circumference	-0.040 (0.635)	0.062 (0.454)	0.044 (0.602)	0.082 (0.328)	0.208 (0.006)	0.257 (0.001)
SBP	-0.051 (0.544)	0.012 (0.887)	0.101 (0.227)	-0.004 (0.961)	0.107 (0.164)	0.101 (0.186)
DBP	-0.099 (0.238)	-0.079 (0.344)	-0.056 (0.500)	0.059 (0.482)	0.137 (0.074)	0.059 (0.443)
FINS	-0.042 (0.612)	-0.085 (0.303)	-0.080 (0.339)	0.013 (0.872)	0.116 (0.128)	0.138 (0.070)
HbA1c	-0.028 (0.738)	-0.149 (0.071)	-0.085 (0.308)	0.001 (0.988)	0.103 (0.177)	0.055 (0.473)
FPG	-0.176 (0.035)	-0.114 (0.169)	-0.036 (0.666)	-0.139 (0.095)	-0.021 (0.790)	0.025 (0.741)
ALT	-0.067 (0.420)	-0.046 (0.580)	-0.067 (0.425)	0.046 (0.579)	0.161 (0.035)	0.207 (0.006)
AST	-0.008 (0.923)	-0.042 (0.614)	-0.060 (0.469)	0.045 (0.591)	0.156 (0.040)	0.199 (0.009)
GGT	0.061 (0.464)	-0.068 (0.413)	-0.148 (0.075)	0.017 (0.835)	0.161 (0.034)	0.139 (0.068)
Scr	-0.050 (0.546)	-0.173 (0.035)	-0.021 (0.798)	0.032 (0.700)	0.085 (0.264)	0.101 (0.188)
UA	0.032 (0.698)	-0.093 (0.260)	-0.140 (0.093)	0.158 (0.057)	0.227 (0.003)	0.310 (0.001)
TC	0.182 (0.028)	-0.016 (0.850)	-0.059 (0.483)	-0.037 (0.656)	0.029 (0.709)	-0.028 (0.719)
TAG	0.051 (0.539)	-0.092 (0.267)	-0.223 (0.007)	0.058 (0.488)	0.027 (0.722)	0.026 (0.732)
HDL-Ch	0.075 (0.366)	0.011 (0.893)	0.098 (0.240)	0.042 (0.619)	-0.024 (0.752)	-0.116 (0.127)
LDL-Ch	0.158 (0.058)	-0.010 (0.907)	-0.076 (0.364)	-0.058 (0.484)	0.068 (0.371)	0.036 (0.638)

### Multiple linear regression analysis of the influencing factors during the subjects' responses

This study adopted a multiple linear regression model to analyse further the response time of the subjects in the behavioural tasks. In the Flanker task, taking the response time of the subjects under consistent stimulation conditions as the dependent variable, the statistically significant indicators and clinically significant indicators in the above partial correlation analysis were included in the model as independent variables. The results revealed that BMI (P=0.0001) was associated with prolonged response time. The response time of the subjects under inconsistent stimulation conditions was taken as the dependent variable for analysis. The results revealed that BMI (P=0.000) and hip circumference (P=0.013) were the influencing factors of long response delay. In the n-back task, the response time of the subjects in the 1-back task was used as the dependent variable, and the statistically significant indicators and clinically significant indicators in the above partial correlation analysis were used as independent variables. The results revealed that BMI (P=0.001) was correlated with the response time of the 1-back task. The analysis was conducted with the reaction time of the subjects in the 2-back task as the dependent variable. The results revealed that BMI (P=0.0001) was also an influencing factor for long reaction delay in the 2-back task. The specific data are shown in Table 5. The above results suggest that BMI is an influencing factor for the response time of the subjects in the flanker and n-back tasks.

**Table 5:** Multiple linear regression analysis of the influencing factors of the reaction time of subjects.

Variable	β	STD	t value	P value
Flanker task				
Incongruent stimulus				
BMI	11.064	0.511	4.053	0.001
Hip circumference	-3.664	-0.315	-2.499	0.013
Congruent stimulus				
BMI	6.468	0.295	4.043	0.001
n-back task				
1-back				
BMI	9.505	0.25	3.378	0.001
2-back				
BMI	15.045	0.317	4.368	0.001

### Correlation analysis of the serum FGF21 levels of the subjects and their performance in behavioural tasks

**Figure 1:**
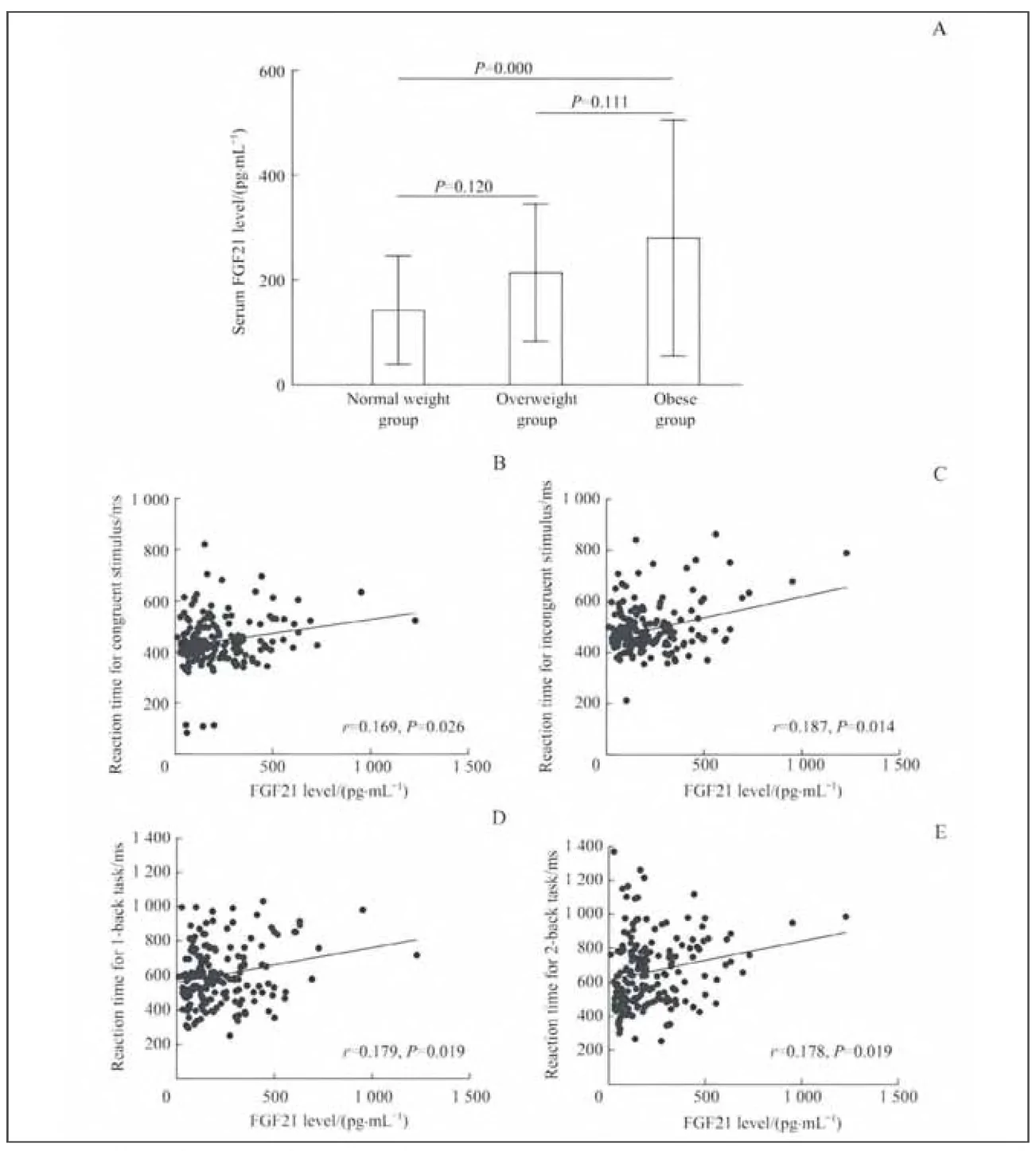
Comparison of serum FGF21 levels among the three groups.

As shown in Figure 1A, compared with those in the normal body type group, the serum FGF21 levels in the obese group were greater (P=0.000). After adjusting for sex and age, we used partial correlation analysis to evaluate the correlation between the reaction time and the serum FGF21 level. The results (Figure 1B-Figure 1E) revealed that the response times of the subjects in the flanker task (under consistent or inconsistent stimulation conditions) and the n-back task (1-back or 2-back task) were positively correlated with their serum FGF21 levels (all P<0.05).

Multiple linear regression analysis was subsequently conducted with the serum FGF21 level as the dependent variable and cognitive function-related indicators as the independent variables. The results (Table 6) revealed that the ACC (P=0.000) of the subjects in the 2-back task and the response time under inconsistent stimulation conditions (P=0.048) were independent influencing factors of their serum FGF21 level.

**Table 6:** Multiple linear regression analysis of cognitive function-related indicators and serum FGF21 levels in subjects.

Variable	β	STD	t value	P value	adjusted R2
2-back ACC	-2.09	-0.334	-3.687	0.001	0.11
Reaction time for an incongruent stimulus	0.002	0.182	2.004	0.048	-

## Discussion

The results of this study revealed that the response time of adolescents in the obese group with respect to basic and higher-order cognitive functions and working memory was significantly greater than that of those in the normal body type group. The BMI of the subjects was positively correlated with their response time in basic and higher-order cognitive functions and working memory, and BMI was an influencing factor for the long response delay in the Flanker task and the n-back task. Furthermore, compared with those of the other two groups, the serum FGF21 levels of adolescents in the obese group were greater. They were significantly correlated with response time in the flanker task and the n-back task.

Previous studies [17] have evaluated the relationship between obesity and the ability to cope with cognitive conflicts in preadolescent children via behavioural tests and neuroelectrical tests. The results showed that under inconsistent stimulation in the Flanker task, the response time of obese children was significantly longer than that of healthy children, suggesting that childhood obesity can reduce their ability to process cognitive conflicts. The relevant neuropsychological test research compared the scores of eight indicators in the CNSVS test between the obese group and the healthy control group of adolescents. The results revealed that the average scores of the obese group of adolescents in all the cognitive domains were significantly lower than those of the control group, indicating that obesity is associated with impairments in multiple aspects of adolescent cognitive function. The results of this study show that, compared with that of the normal body type group, the response time of obese adolescents in the Flanker task under consistent or inconsistent stimulation conditions was significantly prolonged. That is, the performance of obese adolescents at both the basic and higher-order cognitive function levels was worse than that of the normal body type group. This finding may also indicate that obesity has a relatively extensive negative impact on the cognitive function of adolescents.

Weight loss was associated with a reduction in prefrontal lobe activation, indicating that cognitive function changes in obese adolescents are related to the neuroplasticity of metabolic changes [18]. This study revealed that the response time of obese adolescents in both the 1-back and 2-back tasks was significantly longer than that of the normal body type group, indicating that both the immediate memory and delayed memory functions of obese adolescents have declined. Obesity can lead to the accumulation of adipose tissue throughout the body and an increase in the secretion of related cytokines and inflammatory factors, thereby putting obese patients in a state of systemic inflammation [19]. Among them, TAG is believed to mediate the impact of obesity on cognitive function to some extent. For example, high levels of TAG can prevent leptin, FGF21, etc., from passing through the blood brain barrier, thereby adversely affecting the nervous system and ultimately leading to cognitive dissonance. The neurotransmitter dopamine may be the cause of the differences in cognitive function between obese individuals and normal individuals [20]. At present, further research is needed on the changes in cognitive function among obese adolescents. Moreover, more precise methods (such as hydrogen proton magnetic resonance spectroscopy, diffusion tensor imaging, resting-state functional magnetic resonance imaging of the brain, and other emerging magnetic resonance techniques) are needed to assess cognitive function.

The clinical study [21] has shown that obesity and a high-fat diet are associated with deficits in learning, memory and executive functions, as well as potential brain atrophy. A recent study [22] revealed from an epidemiological perspective that excessive obesity is a key metabolic risk factor for cognitive decline. Reducing excessive visceral fat and lowering the waist-to-hip ratio can effectively improve cognitive function and enhance thinking ability, learning ability and memory. Mice fed a high-fat diet have inflammatory damage in the hippocampal region due to liver steatosis, macrophage infiltration, insulin resistance, etc., eventually leading to memory deficits in the mice [23]. Moreover, systemic and central inflammation caused by obesity can affect the function of synaptic neurons through multiple mechanisms, ultimately leading to changes in cognitive function in obese patients. In addition, obesity can also cause damage to the blood brain barrier. Studies [24] have shown that, compared with non-obese rats, rats fed a high-fat diet exhibit increased permeability of the blood brain barrier and hippocampus-dependent cognitive impairment. Moreover, animal studies [25] have shown that after blood brain barrier injury, serum-derived substances can enter the hippocampal space to activate microglia, eventually causing cognitive dysfunction. At present, the pathogenesis of obesity-related cognitive impairment has not been fully clarified and requires more in-depth research and exploration.

FGF21 is a hormone-like protein that is expressed mainly in the liver and can activate downstream signals by forming complexes with fibroblast growth factor receptor (FGFR) and the coreceptor - klotho in target organs. Furthermore, it participates in the body's metabolism and maintains the homeostasis of lipid metabolism and glucose metabolism. Previous studies [26] have shown that the serum FGF21 level in obese patients is significantly greater than that in non-obese individuals. In nonalcoholic fatty liver disease patients with central obesity, the level of this indicator is positively correlated with the liver fat content. This finding is consistent with the results of this study, namely, that the serum FGF21 level of adolescents in the obese group was significantly greater than that in the normal body type group. Furthermore, relevant studies [27] revealed that, compared with those in non-obese mice, the levels of FGF21 in homozygous Lepob mutant mice (ob/ob), Leprdb mutant mice (db/db), and diet-induced obese mice were significantly increased, accompanied by decreased expression of FGFR and - Klotho in target tissues. These findings suggested that the mice were resistant to FGF21. At present, FGF21 has been proven to play an important role in the metabolism and cognition of the brain. Previous studies [14] have shown that FGF21 promotes the expression of brain-derived neurotrophic factors and maintains the stability of mitochondrial proteins by inhibiting the polymerisation of the microtubule-associated proteins tau and -amyloid. It regulates inflammation and oxidative stress through the NF- B (nuclear factor B) and AMPK/Akt (adenosine monophosphate-activated protein kinase/serine-threonine kinase) signalling pathways to alleviate neuroimmune injury, thereby inhibiting neurodegenerative changes. Furthermore, FGF21 can also protect the blood brain barrier by upregulating peroxisome proliferator-activated receptor through FGFR1/ - klotho and reduce the damage caused by inflammation to cognitive function [28][29][30]. Given that the central nervous system may be an important target for FGF21 in the treatment of obesity-related metabolic abnormalities, research on the role of FGF21 in cognitive impairment has significant clinical implications. It may provide new ideas for the diagnosis and treatment of obesity-related cognitive impairment.

## Conclusion

Our study comprehensively evaluated the cognitive function of overweight and obese adolescents through behavioural experiments and revealed that cognitive dysfunction already exists in the obese stage of adolescence. Among them, BMI was an influencing factor for changes in cognitive function in adolescents, and cognitive function was significantly correlated with BMI and FGF21 levels. The research results provide a new direction for further exploration of the mechanism of cognitive changes in obese adolescents.

## Dodatak

### Availability of data and materials

The datasets generated or analysed during the current study are not publicly available because they contain private information. However, the data are available from the corresponding author upon reasonable request.

### Authors' contributions

All the authors contributed to editorial changes in the manuscript. All the authors read and approved the final manuscript. All authors have participated sufficiently in the work and agreed to be accountable for all aspects of the work.

### Conflict of interest statement

All the authors declare that they have no conflict of interest in this work.
